# A Global Industry Survey on Post-Approval Change Management and Use of Reliance

**DOI:** 10.1007/s43441-024-00681-y

**Published:** 2024-08-23

**Authors:** Andrew Deavin, Aliyah Hossain, Isabelle Colmagne-Poulard, Kum Cheun Wong, Mónica Perea-Vélez, Sonia Cappellini, Susanne Ausborn, Sylvie Meillerais, Céline Bourguignon

**Affiliations:** 1 GSK, 20 Avenue Fleming, 1300 Wavre, Belgium; 2 GSK, GSK House, 980 Great West Road, Brentford, Middlesex TW8-9GS UK; 3 Merck KGaA, 1 Route de Crassier, 1262 Eysins, Switzerland; 4 Novartis Asia Pacific Pharmaceuticals Pte Ltd, 20 Pasir Panjang Road, #10-25/28, Mapletree Business City, 117439 Singapore; 5 Menarini Ricerche S.p.A, Via Tito Speri 10, 00071 Rome, Italy; 6 F. Hoffmann–La Roche Ltd, Grenzacher Strasse 124, 4070 Basel, Switzerland; 7 MSD Europe Belgium SRL, Boulevard du Souverain 25, 1170 Brussels, Belgium

**Keywords:** Post-approval changes (PACs), Reliance, ICH Q12, Pharmaceutical supply, Regulatory convergence

## Abstract

Post-approval changes (PACs) to the control and manufacturing processes of medicines and vaccines are routinely undertaken and critical to enable both innovation and secure sustained supply. In a world of global supply chains, the existence of divergent national PAC requirements (with additional countries introducing new requirements with potential differences) and other factors including document preparation and response timelines, can lead to long delays in approval (of up to 3–5 years) increasing the risk of disruption and shortages.

We undertook an Industry survey in 2023 to assess implementation of ICH Q12, PAC procedures (change categorisation and review timelines) and use of reliance mechanisms across different countries (9 selected ICH Members and 19 Observers). Although this survey revealed limited implementation of Q12 in ICH Member countries, when comparing the data collected with those of a previous survey performed in 2020, we observed a broader adoption of risk-based approaches to variation categorisation (in all countries). This, however, was not reflected in improved timelines for approval.

With regards to ICH Q12 adoption, the uptake of Post-Approval Change Management Protocols (PACMPs) was unchanged (with only one country reporting in-use) and implementation gaps were evident for Established Conditions (EC) and the Product Life Cycle Management document (PLCM). The survey found greater awareness of ICH Q12 and its tools compared to 2020, potentially illustrating the positive impact of training efforts. This illustrates the challenges being faced to broaden its implementation and use globally.

In the same Industry survey, we also assessed PAC processes across different international countries. Long unpredictable timelines were the major concern across the countries surveyed together with limited capacity of the regulators. Four different CMC changes were selected and categorized by the respondents according to current knowledge of national classifications and timelines in the selected countries and compared with a reference classification and timeline from the European Medicines Agency and the World Health Organisation. This highlighted the lack of harmonisation of many countries with EU/WHO requirements, especially within the ICH Observer group.

Last, this survey showed that some use of unilateral forms of reliance to Reference Authorities for PACs is starting. This is a mechanism all countries can employ, regardless of convergence of requirements and expertise, to enhance capacity building and reduce duplication of reviews, streamline variations approval, whilst accelerating patient access to innovation and securing supply.

**Supplementary Information:**

The online version contains supplementary material available at 10.1007/s43441-024-00681-y.

## Introduction

Post-approval changes (PACs) to the control and manufacturing processes of medicines and vaccines, are routine and critical to enable both innovation and secure sustained supply. They are needed to enhance the robustness and efficiency of the manufacturing process, facilitate supply, improve quality control techniques, respond to changes in the regulatory requirements and upgrade to state-of-the-art facilities. However, in a world of global supply chains, the existence of divergent national PAC requirements (with additional countries introducing new requirements with potential differences) and other factors including document preparation and response timelines, can lead to long delays in approval (of up to 3–5 years) [1, 2] increasing the risk of disruption and shortages.

In recent years, more countries have introduced a national PAC regulatory framework with their own requirements and timelines. The International Council for Harmonisation of Technical Requirements for Pharmaceuticals for Human Use (ICH) Q12 guideline (on technical and regulatory considerations for pharmaceutical product lifecycle management, 2020) was introduced to establish tools and principles for science- and risk-based approaches, with the aim of supporting the harmonisation of the PAC systems globally [3]. However, in the last Centre for Innovation in Regulatory Science (CIRS) survey of industry and regulators conducted in 2021 [4], ICH Q12 was either ‘not implemented’ or ‘being implemented’ (n.b. there is no implementation timeline for ICH Tier 3, which includes ICH Q12). In parallel, efforts are underway to use reliance mechanisms for PACs, in part triggered by the Covid-19 pandemic in 2020/21 where changes to medicinal products needed to be executed quickly and harmoniously. This has led to a recent initiative in 2022 by the International Coalition of Medicines Regulatory Authorities (ICMRA) concerning two pilot programmes focusing on hybrid inspections and collaborative assessments of PACs [5].

Despite these positive developments in the landscape, significant challenges still exist namely: inconsistent classification, additional data requests, unpredictable timelines, divergent decisions and variable post-change implementation periods. These have been highlighted, together with proposed solutions, in an Industry perspective on PACs [6].

We wanted to obtain a better understanding of the remaining challenges experienced by Industry. As ICH Q12 implementation is intended to provide ‘a framework to facilitate the management of post-approval CMC changes in a more predictable and efficient manner’, we started by comparing ICH Q12 implementation progress against a similar survey conducted amongst EFPIA (European Federation of Pharmaceutical Industry Associations) members in 2020 [7]. Then, using the key recommendations for PAC regulatory frameworks described in the above publication [6], we assessed the relative impact of these challenges on PAC processes, compared PAC categorisations and timelines, and identified reliance mechanisms available for PACs.

Based on the data gathered, we propose the next steps needed to drive simplification of life cycle-management and facilitate continuous supply of medicinal products worldwide.

## Method

Further details on the methodology can be found in the Annex.

### First ICH Q12 Survey (2020)

The first survey assessed the following countries that were ICH Regulatory Members (hereafter referred to as **‘ICH Members’**) at the time: Brazil, China, Singapore, South Korea, Chinese Taipei, Turkey. Note: USA, EU, Japan, Canada and Switzerland were not included as we wanted to focus on the emerging key regulatory members in ICH [8].

We utilized regional and/or country networks of Regulatory Experts from the European Federation of Pharmaceutical Industry Associations (EFPIA) covering the major regions (outside Europe and North America) to consolidate input into the survey. EFPIA currently has 33 member companies [9]. Each network is composed of regulatory experts from, on average, 20 different pharmaceutical companies (range 12–30 members).

The survey was limited to questions on whether a risk-based categorisation system for PACs was in place, if Established Conditions (ECs), Post-Approval Change Management Protocols (PACMPs) and Product Life-Cycle Management documents (PLCMs) could be used in line with ICH Q12 and if further education was needed to help implement.

### Current ICH Q12 and PACs Survey (2023)

In the second survey, two groups of countries were selected for assessment:

Selected (International) **ICH Member Countries:** Original six countries (as above) plus Egypt, Mexico and Saudi Arabia [8].

Nineteen (19) Selected ICH Regulatory Observer Countries and members of ICMRA (International Coalition of Medicines Regulatory Authorities) (hereafter referred to as **‘ICH Observers’**): Algeria, Argentina, Australia, Azerbaijan, Colombia, Cuba, Ghana, India, Indonesia, Iran, Israel, Jordan, Lebanon, Malaysia, Moldova, New Zealand, Nigeria, South Africa, and Tunisia [8, 10].

We again utilized the networks of Regulatory Experts from EFPIA covering the major regions outside Europe and North America. In addition, industry members from the International Federation of Pharmaceutical Manufacturers and Associations (IFPMA) provided expertise for Africa.

The data collection took place from April 2023 to June 2023.

#### ICH Q12 Implementation

We first looked at the implementation of ICH Q12 in the selected ICH Members only. We asked the same questions in 2023 as in 2020 enabling us to compare the results and assess any changes.

#### General Assessment of PACs (Timelines and Classification of Four Typical Changes)

The second part of the survey focused on a general assessment of PACs by examining their classification, barriers to implementation, and potential use of reliance. For this assessment both the Selected (International) ICH Member Countries and Selected ICH Observer Countries were surveyed. We asked respondents to pick the top two major issues that caused delay/slow approval or implementation of post approval changes. Then, to study the range of assessment timelines and variation classifications, we selected four different changes that attempted to represent the array of modifications that could be made to a product licence. Respondents were asked to categorise the regulatory action for each change (for both Small Molecules and Biologicals) based on current knowledge and how it would be managed in a real situation. The expected classification and typical time periods (in months) were recorded. These are described further below.

Assessed changes:

- Specification change (tighten) to the drug substance or drug product
- Specification change (widen) to the drug substance or drug product
- Manufacturing change to the drug substance e.g. change to synthesis or additional manufacturing step
- New drug product formulation facility (new site)

Respondents were asked, based on experience and internal company data, to collectively assign one of the following classifications and typical time periods (in months) for each of the above changes (for both Small Molecules and Biologicals):

- **Classification**
  o Tell, wait for formal approval and then do
  o Tell, wait for a defined period e.g. 30 days, then do
  o Do (implement the change) and tell (inform the regulator)
  o No report/regulatory action needed
- **Typical assessment time** (months) from submission to regulatory approval or notification (0, 1, 1 to 3, 3 to 6, 6 to 12, 12 to 18, > 18). This includes the question and response time-period and was based on experience and planning of these variations in the countries.

These two parameters were combined in ‘bubble’ graphs where the size of the bubble indicated the number of countries with that combination of classification and assessment time. We then used the WHO and EMA classification and recommended timeline for each change as a reference point (see Annex) [11–14]. It is important to note that for the assigned reference classifications we assumed that all conditions would be met.

#### Reliance Assessment

Finally, to assess the use of reliance we asked all respondents (countries belonging to ICH Regulatory Member and Observer groups) if any form of reliance had been leveraged to approve PACs and classified the different types used (reliance, unilateral recognition, worksharing, regional reliance and mutual recognition) based on definitions in WHO’s guideline on Good Reliance Practices [15].

## Results

### Assessment of Implementation of ICH Q12 (selected ICH Member Group only)

We first assessed the implementation of ICH Q12. All surveyed ICH Member countries had some type of risk-based PAC classification in place (compared to 2020, when only half did).

However, in contrast to the variation classification, there was little evidence of adoption of the ICH Q12 risk-based tools. No country in scope had implemented ECs or the PLCM document at the time of the survey.^1^ Moreover, other than Brazil, there was no use of PACMPs across the countries surveyed. In Brazil use of PACMPs was recorded in both periods for drug substance (small molecule) only, with an introduction of a pilot for Drug Product in 2023.

We analysed the perceived level of understanding across the country grouping. The number of countries with limited understanding of ECs had decreased compared to 2020 (Figure in Annex). This was consistent for all three tools, indicating there is a greater awareness of ICH Q12 in most countries paving the way for a smoother implementation of the guideline once further advanced ICH training is available.

### General Assessment of PACs (ICH Member and ICH Observer Groups)

For the ICH Observer group, the top 2 barriers perceived to cause delays or slow approvals of PACs were long unpredictable timelines and capacity of NRAs (which relates to human resource with the appropriate expertise). Responses related to the group of selected ICH members also highlighted long unpredictable timelines alongside additional local requirements. This is despite a risk-based PAC classification being in place (see above).

**Figure 1.**
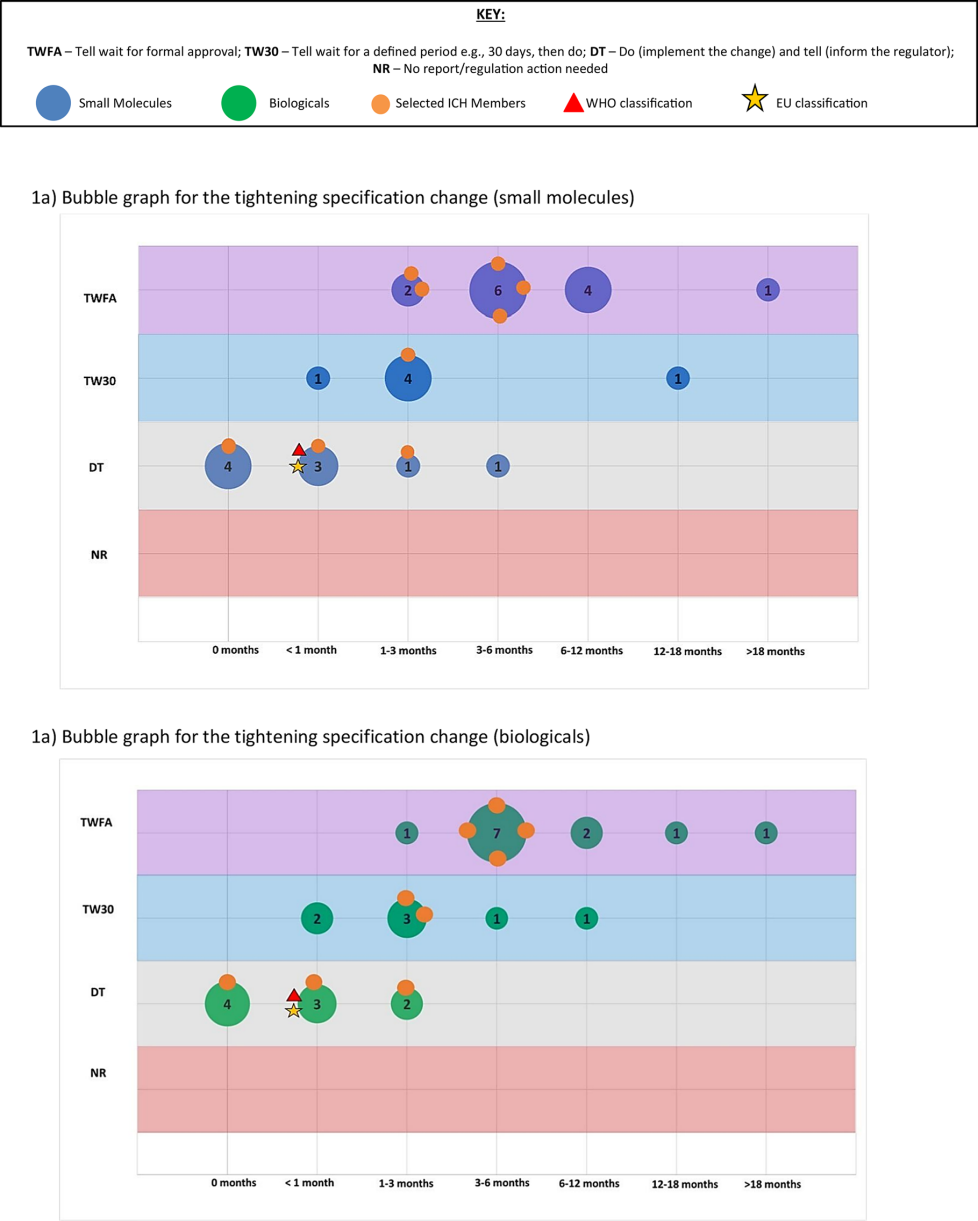

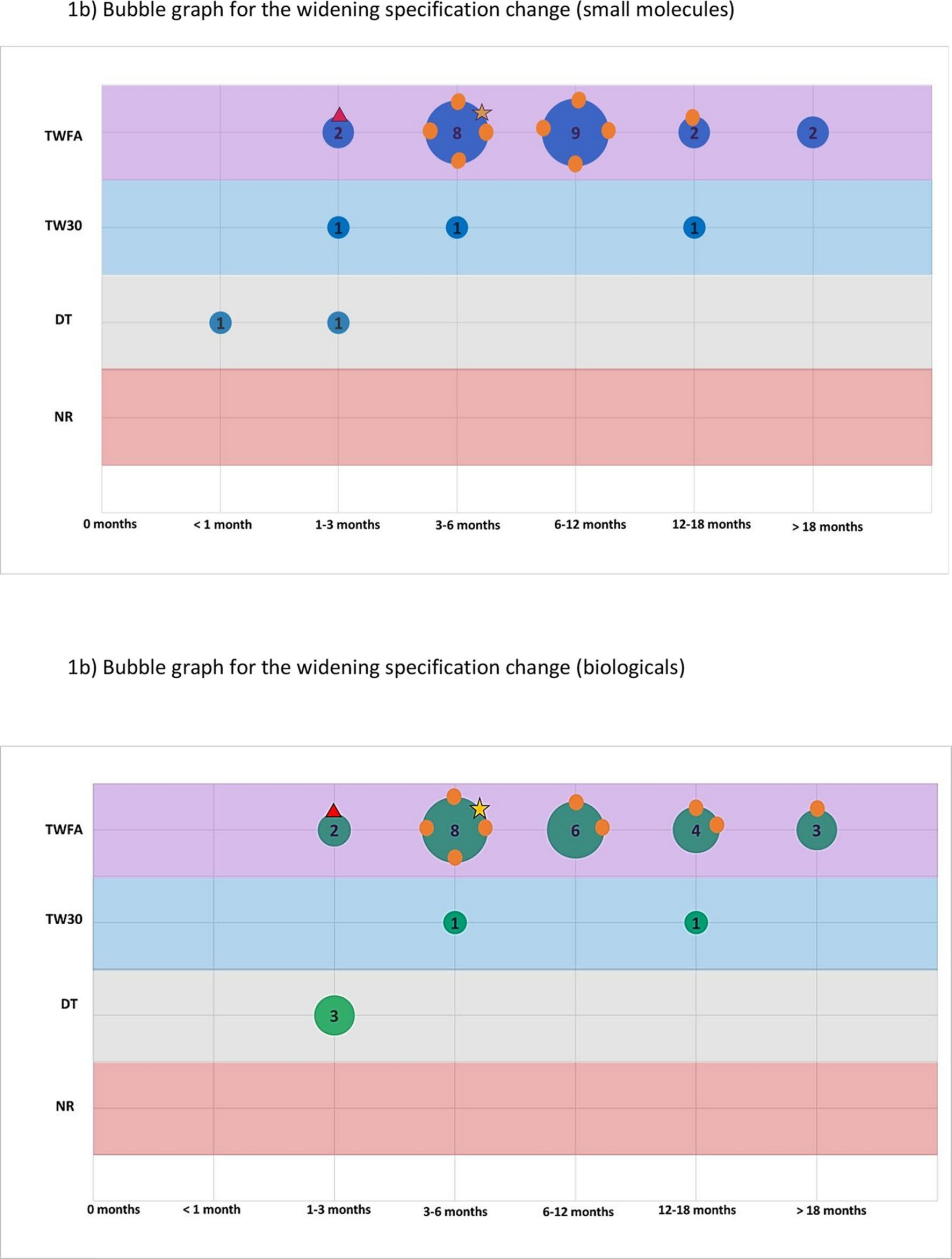

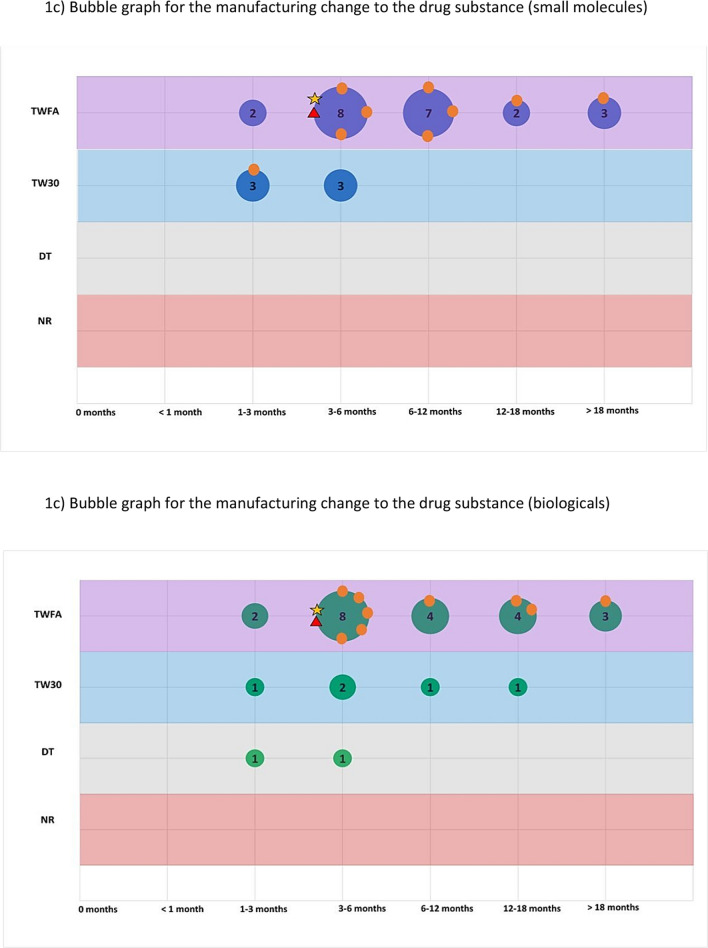

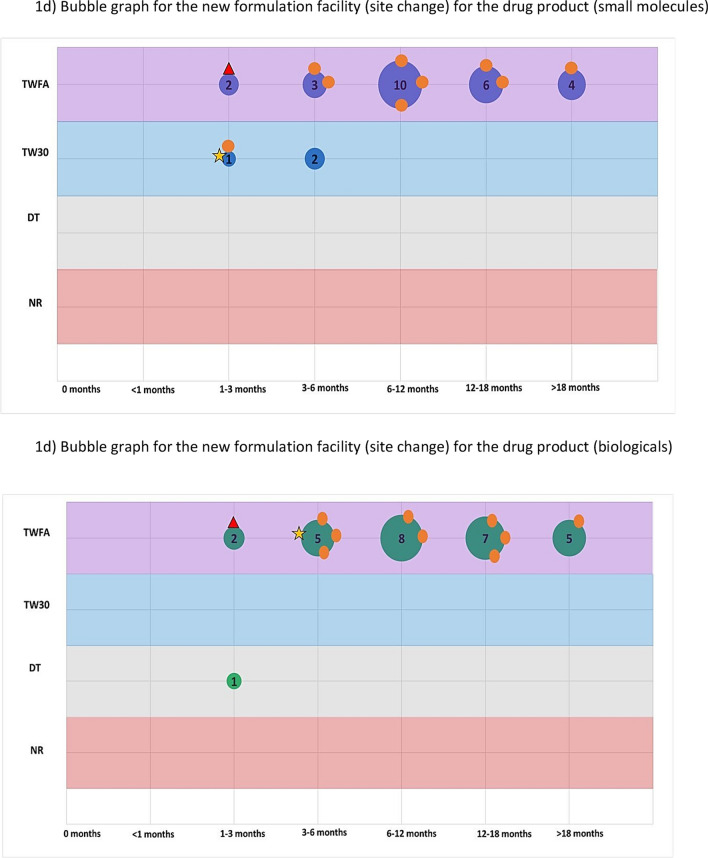
A series of bubble graphs showing the range of classifications and timelines for each selected change (**a**) narrowing of a specification, (**b**) widening of a specification, (**c**) drug substance manufacturing change and (**d**) a new drug product formulation facility. The bubble size represents the number of countries assigned with a classification and timeline; the selected ICH member countries are also specifically highlighted. The reference classification and timeline for WHO and EMA are also plotted on the graph.

This is further illustrated in Fig. 1 which shows, for 4 selected changes, that there is considerable variability in the variation timelines and assignment of categories for each country. The size/number present on the bubble represents the number of countries and the orange circles represent where the ICH member countries are on the graph in comparison to the EU/WHO benchmark.

The results for a variation to tighten a specification are shown in Fig. 1a and Table 1. For both small molecules and biologicals, most countries specified that the classification of the changes was more stringent than the EMA and WHO benchmark point and the timelines were generally longer. Most selected ICH member countries were also clustered at higher classifications than the benchmark. Based on a visual comparison, no clear difference was observed between small molecules and biologicals; there appeared more variability overall for this change in comparison to the other three changes. As shown in Table 1, only 3 countries aligned with the benchmark with most countries in both ICH groups displaying a higher classification and longer timelines than the reference. Of note, no country classified the change as ‘no report/regulatory action needed’; this observation is also relevant for all changes assessed in the survey.

**Table 1.** A tabulation of the results of the classifications and timeline assessment for each selected change. The table shows the results for small molecules (a) and for biologicals (b). The table compares the results from the selected ICH Members and selected ICH Observers and alignment of timelines and classification against the WHO and EMA reference.

	Specification change (tighten) to the drug substance or drug product	Specification change (widen) to the drug substance or drug product	Manufacturing change to the drug substance e.g. change to synthesis or additional manufacturing step	New drug product formulation facility (new site)
*(a) Small molecules*				
EU/WHO Benchmark Reference				
Time range of variation	< 1 month	**EU**: 3–6 months	3–6 months	1–3 months
		**WHO:** 1–3 months		
Classification of change	Do (implement the change) and tell (inform the regulator)	Tell, wait for formal approval and then do	Tell, wait for formal approval and then do	**EU:** Tell, wait for a defined period
				**WHO:** Tell, wait for formal approval and then do
ICH Members (n = 9)				
Aligned with benchmark	1	4 (EU) 0 (WHO)	3	1 (EU) 0 (WHO)
Not aligned with benchmark	8	5 (EU) 9 (WHO)	6	8 (EU) 9 (WHO)
ICH Observers (n = 19)				
Aligned with benchmark	2	4 (EU) 2 (WHO)	5	0 (EU) 2 (WHO)
Not aligned with benchmark	17	15 (EU) 17 (WHO)	14	19 (EU) 17 (WHO)
*(b) Biologicals*				
EU/WHO Benchmark Reference				
Time range of variation	< 1 month	**EU**: 3–6 months	3–6 months	**EU**: 3–6 months
		**WHO:** 1–3 months		**WHO:** 1–3 months
Classification of change	Do (implement the change) and tell (inform the regulator)	Tell, wait for formal approval and then do	Tell, wait for formal approval and then do	Tell, wait for formal approval and then do
ICH Members (n = 9)				
Aligned with benchmark	1	4 (EU) 0 (WHO)	5	3 (EU) 0 (WHO)
Not aligned with benchmark	8	5 (EU) 9 (WHO)	4	6 (EU) 9 (WHO)
ICH Observers (n = 19)				
Aligned with benchmark	2	4 (EU) 2 (WHO)	3	2 (EU) 2 (WHO)
Not aligned with benchmark	17	15 (EU) 17 (WHO)	16	17 (EU) 17 (WHO)

The contrasting variation to widen a specification is shown in Fig. 1b and Table 1. It should be noted that the EMA reference point is different for both small molecules and biologicals based on timelines but not classification. For both small molecules and biologicals, most countries recorded a more stringent classification than the benchmark. Moreover, the timelines were much longer with half the countries taking between 6–12 months and > 18 months to implement the change. Whilst the classification and timeline of 4 ICH members aligned with the benchmark (EMA), 5 had longer timelines for the same change and none aligned with the WHO reference. This was further pronounced when assessing the ICH Observer group where the majority did not align with either reference and, whilst some adopted shorter timelines and lower classifications, more than half the group had longer timelines. There was no clear difference in the visual pattern between small molecules and biologicals.

The results for manufacturing process changes to the drug substance are shown in Fig. 1c and Table 1. For both small molecules and biologicals, the change classification specified by most countries coincided with the EMA and WHO benchmark though timelines were longer frequently extending from 6 months to greater than 18 months compared to 3–6 months. When looking at the two groups of countries, most ICH member countries aligned with the EMA and WHO (for small molecules 3 countries and for biologicals 5 countries) whereas most countries in the ICH observer did not align. When comparing small molecules and biologicals, more variability and spread overall for biologicals was observed with few countries classifying the change as less stringent than the EMA/WHO benchmark.

The results related to a drug product formulation facility change is shown in Fig. 1d and Table 1. As for the widening specification change, it should noted that the EMA and WHO reference points are slightly different for both small molecules and biologicals. As shown in Table 1, few countries aligned with the benchmark with most adopting a higher classification and longer timelines. The same pattern was seen for both small molecules and biologicals and no discernible difference between the two country groupings based on a visual comparison, with only a minority of the ICH Members clustering with the EMA or WHO reference.

Overall, the results showed that longer timelines and greater stringency would be expected by the respondents for the four changes evaluated across all countries assessed, compared to the EMA/WHO reference. This was most pronounced in the selected ICH Observer group.

### Reliance Assessment (ICH Member and ICH Observer Groups)

**Figure 2.**
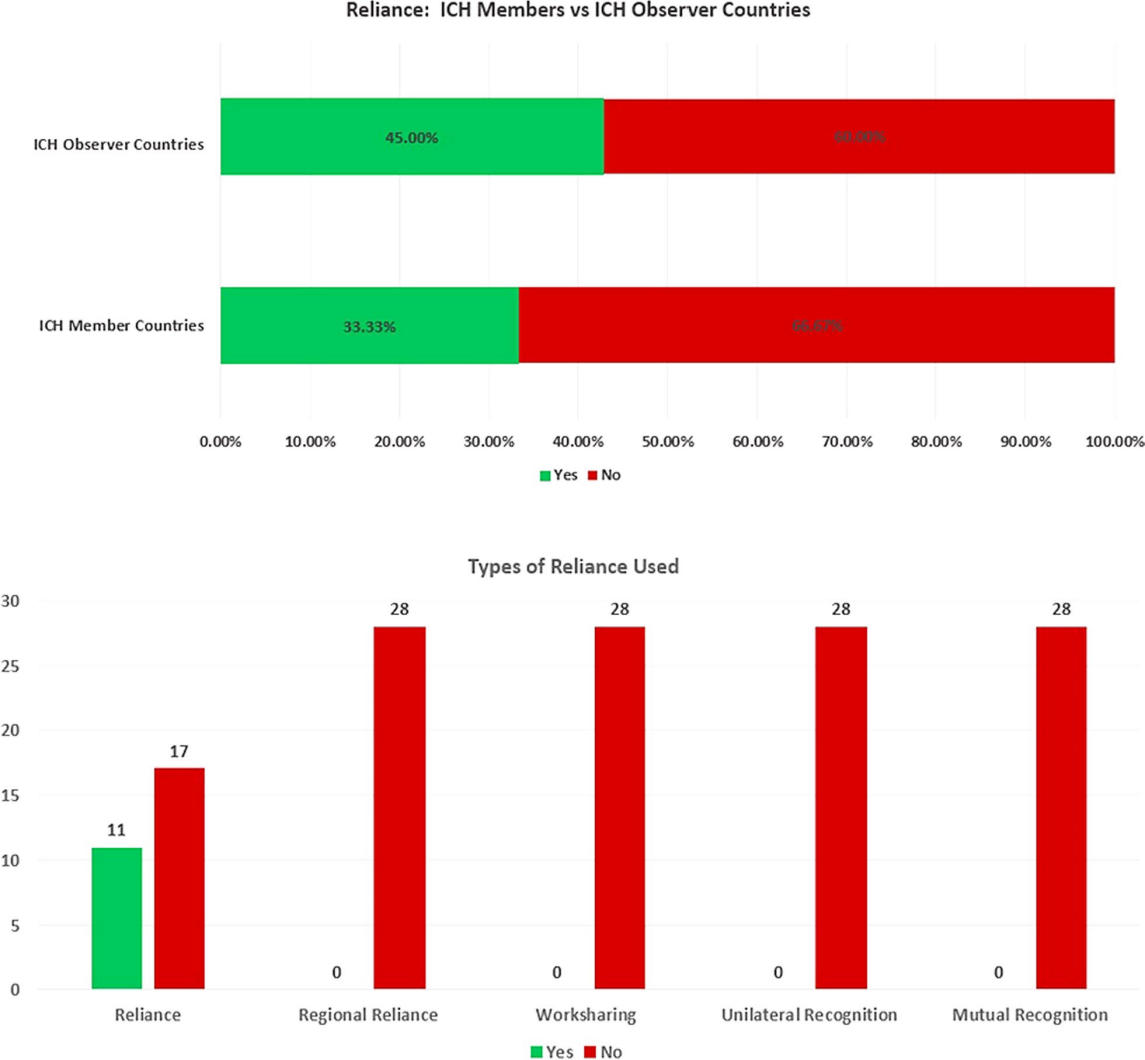
Shows the difference between ICH Member Countries and ICH Observer Countries and their use of reliance in the assessment and approval of PACs. It also shows the types of reliance mechanism (as per WHO definition [13]) used by all countries in the assessment and approval of PACs.

Whilst reliance is not used routinely for the assessment or approval of PACs, 1/3 of countries had used some form of reliance, e.g., to reduce variation backlog. The use of reliance was more prominent in the ICH Observer countries as shown in Fig. 2, with few ICH Member countries using this approach. Where reliance was used, only the basic reliance mechanism (as defined in the methodology ‘… the regulatory authority in one jurisdiction takes into account … assessments performed by another regulatory authority … in reaching its own decision.’) [15] was employed. In contrast, the more complex reliance mechanisms, such as worksharing, regional reliance, unilateral recognition and mutual recognition, were not used (Fig. 2).

## Discussion

This survey assessed the implementation of ICH Q12 tools in selected ICH countries and identified barriers to PAC systems from an Industry perspective. By evaluating selected ICH Member countries with ICH Observer group, we were able to assess and compare countries of differing regulatory maturity.

### Implementation of ICH Q12 in the ICH Member Countries

We specifically looked at the impact of ICH Q12 implementation in the selected ICH Member countries. As for all ICH members, this group is expected to implement ICH Q12 but, to date, this has been slow to happen. There is a high level view of ICH Q12 implementation on the ICH website [16] which includes the status for founding regulatory members. According to the ICH website the following countries have implemented ICH Q12 (as of 17 June 2024): USA FDA (May 21), Japan PMDA (October 21) and China NMPA (which initiated a pilot in August 23, after the survey period had completed [17]); the other ICH member countries are in the process of implementation or have not yet implemented.

Our results and the latest status on the ICH website are similar to findings in the CIRS ICH report (2021) [4] on the adequacy of implementation and adherence to ICH regulatory guidelines by regulatory authorities. CIRS collected responses from both industry and regulators and found that ICH Q12 (a Tier 3 guideline) was perceived to be ‘in the process of being implemented or not implemented’ by the respondents (n.b. no authority considered the guideline implemented). CIRS has initiated another similar survey of regulators and industry, the output of which will be interesting to determine if there has been an improvement in implementation when it is published later in 2024.

In our industry survey, we looked specifically at the different science- and risk-based tools described in ICH Q12. In doing so, we observed a positive trend with the adoption of risk-based approaches to variation categorisation in all countries; this is an improvement compared to our 2020 survey of the EFPIA Network members. When comparing the variable categorisation of PACs (see Results, Fig. 1), it is evident that there is still room for further maturity here even amongst the selected ICH members.

PACMP is a valuable tool to enable prior approval of the methodology before accelerated approval of the technical results which can be applied to certain change types like a new assay or even a site change. This tool, which was introduced in earlier WHO guidelines as well as ICH Q12, can contribute to reducing variation timelines by using an appropriate reporting category, enhancing clarity of the evidence needed in support of the change and building trust between agencies and industry. It was therefore disappointing to observe limited uptake of PACMPs compared to 2020. One reason for this may be due to the need for legislation changes to allow for a reduction in regulatory classification for submission of the results once the strategy has been approved in the PACMP. Such legislation changes are often complex to adopt and take time to come in effect.

The lack of implementation of ECs and the PLCM was expected by the authors given the slow implementation globally. We anticipate that USA, Europe and Japan will first need to share their experience and lessons learnt before further wider adoption. It is important to note that, shortly after we conducted the survey, China regulators announced an ICH Q12 pilot which could open the way for applications with ECs [16]; this is an important development and its successful implementation could help to broaden its use.

Finally, our data show that there is now greater awareness of ICH Q12. This shows the positive impact that training efforts are having; it is a key step to broader introduction and use of risk-based approaches not just for change classification but also for all the science- and risk-based tools needed for full implementation of ICH Q12.

### Approval Timelines for Changes

The findings indicate that long unpredictable timelines are the major concern for Industry across the countries surveyed with most countries exhibiting longer assessment timelines and higher categorization than the EMA/WHO reference. Indeed, the real picture is a staggered effect spreading over months or even years, where applicants submit at different time periods in multiple countries. Consequently, whilst the graphics present a simultaneous picture, the reality is a protracted period from the first to the last approval spanning several years and involving other changes submitted and approved in the same staggered manner globally. In a recent publication of Pfizer data covering the period 2016–2020 (over 790 CMC changes across multiple products, in over 3,575 country submissions across 97 countries) it was shown that the time to achieve a 90% probability of approval for a change was ≥ 24 months in 63% of countries studied and ≥ 36 months in 15% of countries studied [1]. Similarly, a study which analysed over 145,000 PACs covering 156 countries (collected by 18 global pharma companies over a 3-year period, 2019–2021) found that only 1 of the country National Regulatory Authorities (NRAs) approved all submitted PACs within a period of 6 months. Moreover, in 33 countries more than half of the PACs assessed took more than 6 months for approval [2].

Limited capacity of the regulator and the greater number of PACs submitted in recent years could lead to longer approval timelines observed in the survey. This was the second major concern cited by Industry in our survey, primarily highlighted in the ICH Observer group. This can be addressed though further education on new technical developments with shared training and knowledge exchange between regulators. In addition, reliance mechanisms could be helpful in enhancing capacity building as well as targeting the regulator’s resource by minimising duplicative reviews (see below).

### Comparison of Timelines and Classification with the EMA/WHO Reference

This survey design was able to compare selected ICH Member and Observer countries. We saw that the ICH Member group was closer to the EMA/WHO reference in terms of timelines and categorisation than the Observer country group; closer alignment though is still needed. Similar disharmonisation was also reported in a recent article that compared PAC classifications in Latin American countries with that in EU and USA [18]. Globally all countries need to align more closely with reference to science- and risk-based classifications and timelines, thus reducing additional and variable local requirements. These factors (i.e. harmonization of classification and timelines) are drivers for expanding reliance which will, in turn, reduce global submission and approval timelines, increase predictability and improve the supply of medicinal products to patients.

More variability was observed in classifications and timelines for the manufacturing process change and new drug product formulation facility for biologicals, but surprisingly we saw similar patterns for both small molecules and biologicals for tightening and widening of specifications. In our experience, Regulators tend to establish classifications and timelines for small molecules first before tackling biologicals. If clear guidelines are lacking, then applicants may justify that no regulatory action is needed or alternatively class as prior approval variations with no option to file a notification or moderate change.

### Reliance Mechanisms for PACs

It is encouraging to see the increasing use of unilateral reliance. This is a mechanism all countries can employ regardless of the level of convergence (of requirements). Leveraging a reference agency assessment can help to accelerate the approval of variations in each country and therefore we can see significant opportunity for nations to employ reliance more readily in both groups that we studied. This is illustrated by recent pilots using reliance for post-approval changes [4], such as a report on a procedure across 21 national regulatory authorities after initial approval was granted by a reference authority [19]. The authors highlight the opportunities and challenges of implementing reliance principles for post-approval changes, including engaging the reference authority and the NRAs.

So how can reliance practices be more widely implemented especially for life-cycle management? In our view, industry and regulators need to explore together greater opportunities to utilise reliance across the life cycle.

More specifically and from our experience, relying regulators want to verify that the change is approved by the reference authority and, in addition, confirm that what the applicant has submitted is the same as received by the reference agency. Mechanisms need to be adopted to fully address this. For example, clearer reference to the precise change in the approval letter or other documentation could be given by the regulator and the applicant could provide proactive justification of any differences between the reference agency and relying agency information.

More broadly, regulatory convergence is needed globally. Differences in regulatory frameworks in initial and post-approval stages worldwide, present challenges for reliance and should be addressed to simplify the process for users. Finally, stakeholders should evaluate the lessons learned from reliance pilots, develop best practices to standardize the approaches and develop metrics to measure implementation of reliance. Together these measures, could help to further encourage the wider adoption of reliance which we hope will be the routine and standard regulatory mechanism for initial and post-approval applications.

## Conclusion

We consider this to be the first survey to assess the timeline and classification of different changes and the use of reliance for PACs across a broad cross-section of industry. Additionally, it uniquely provides a greater understanding of the ICH Q12 tools for science- and risk-based approaches implementation.

By focusing on only four changes representing a range of changes and a standardized classification of reliance based on WHO and EMA criteria, we have been able to obtain global footprints for 2023 that can be used to compare in the future and show evolution over time. The same can be done for the ICH Q12 tools; indeed, we were able to reference earlier data collected in 2020 to compare with that obtained in 2023. We could therefore also consider investigating, in the future, specific use cases of ECs and PACMPs. Taken together this survey provides a unique insight into the global PAC landscape by highlighting the challenges faced by applicants when submitting PACs.

In conclusion, this methodology and approach can be used to measure the convergence of PAC systems, highlight the gaps to trigger improvement and in future show closer alignment of regulatory timelines, risk-based categorization and wider use of reliance over time. It is important to note that the joint work plan to support a global regulatory Pharmaceutical Quality Knowledge Management (PQKM) capability^2^ [20] also focuses on similar factors of: aligning on data elements and standards; alignment of regulatory assessment and expectations; and cross-organisational collaboration. Ultimately closer alignment will lead to faster global regulatory approvals with similar regulatory outcomes in the same timeframe, which will be highly beneficial to patients by accelerating access and improving supply.

## Supplementary Information

Below is the link to the electronic supplementary material.

Supplementary file1 (DOCX 77 KB)

## Data Availability

Data is provided within the manuscript or supplementary information files.

## Footnotes

1 NMPA released the official notice announcing the implementation of ICH Q12 in China effective from August 25,2023. This includes a 24-month transition period for ICH Q12 trial implementation (starting from 25 August 2023).

2 Developed by International Coalition of Medicines Regulatory Authorities (ICMRA), International Council for Harmonisation of Technical Requirements for Pharmaceuticals for Human Use (ICH), International Pharmaceutical Regulators Programme (IPRP), and Pharmaceutical Inspection Co-operation Scheme (PIC/S).
