# Correction: A Global Industry Survey on Post-Approval Change Management and Use of Reliance

**DOI:** 10.1007/s43441-024-00733-3

**Published:** 2024-12-28

**Authors:** Andrew Deavin, Aliyah Hossain, Isabelle Colmagne-Poulard, Kum Cheun Wong, Mónica Perea-Vélez, Sonia Cappellini, Susanne Ausborn, Sylvie Meillerais, Céline Bourguignon

**Affiliations:** 1 GSK, 20 Avenue Fleming, Wavre, 1300 Belgium; 2 GSK, GSK House, 980 Great West Road, Brentford, Middlesex TW8-9GS UK; 3 Merck KGaA, 1 Route de Crassier, Eysins, 1262 Switzerland; 4 Novartis Asia Pacific Pharmaceuticals Pte Ltd, 20 Pasir Panjang Road, #10-25/28, Mapletree Business City, 117439 Singapore; 5 Menarini Ricerche, S.p.A, Via Tito Speri, 10, 00071 Rome, Italy; 6 F. Hoffmann–La Roche Ltd, Sylvie Meillerais, Grenzacher Strasse 124, Basel, 4070 Switzerland; 7 MSD Europe Belgium SRL, Boulevard du Souverain 25, Brussels, 1170 Belgium

**Therapeutic Innovation & Regulatory Science (2024) 58:1094-1107**

10.1007/s43441-024-00681-y

The article “A Global Industry Survey on Post-Approval Change Management and Use of Reliance,” by Andrew Deavin, Aliyah Hossain, Isabelle Colmagne-Poulard, Kum Cheun Wong, Mónica Perea-Vélez, Sonia Cappellini, Susanne Ausborn, Sylvie Meillerais, and Céline Bourguignon, was originally published was originally published electronically on the publisher’s internet portal on August 23 2024 without open access. With the author(s)’ decision to opt for Open Choice the copyright of the article changed on December 3 2024 to © The Author(s) 2024 and the article is forthwith distributed under a Creative Commons Attribution CC BY.

