# Supplementary material for: A Global Industry Survey on Post-Approval Change Management and Use of Reliance
Source: Ther Innov Regul Sci. 2024 Aug 23;58(6):1094–107. doi: 10.1007/s43441-024-00681-y (PMC11682004; doi:10.1007/s43441-024-00681-y)
Supplement: Supplementary file 1 — Supplementary file1 (DOCX 77 KB) [file 43441_2024_681_MOESM1_ESM.docx]

# Supplementary Information

**Further Details on Methodology**

*General Assessment of PACs (timelines and classification of four typical changes)*

To analyse the barriers to PACs we asked respondents to pick the top two major issues that caused delay/slow approval or implementation of post approval changes at the time of the survey. The issues were based on free-text responses obtained in an earlier survey of EFPIA members in 2020 (internal data). By forcing respondents to pick only two issues we hoped to get clear prioritisation of the major issues facing applicants submitting PACs. To capture any other barriers that were not listed, we also included a free text ‘other’ field.

*WHO and EMA Reference Classification*

For the assessment of variation timelines and assignment of categories of four selected changes, we compared to a WHO and EMA reference. The tables below show the assignments and references that are displayed in the graphs (Figure 1).

***Annex Table:*** *Table showing the time ranges and classifications for each change assessed for EU and WHO, together with the reference for each classification.*

**SMALL MOLECULE**

|  | **Specification change (tighten) to the drug substance or drug product** | | **Specification change (widen) to the drug substance or drug product** | | **Manufacturing change to the drug substance e.g. change to synthesis or additional manufacturing step** | | **New drug product formulation facility (new site)** | |
| --- | --- | --- | --- | --- | --- | --- | --- | --- |
|  | Time range of variation | Classification of change | Time range of variation | Classification of change | Time range of variation | Classification of change | Time range of variation | Classification of change |
| **EU** | < 1 month | do (implement the change) and tell (inform the regulator) | 3-6 months | tell, wait for formal approval and then do | 3-6 months | tell, wait for formal approval and then do | 1-3 months | tell, wait for a defined period, then do |
| **Reference** (2013/C 223/01) | B.I.b.1. b) and B.II.d.1 a) | | B.I.b.1. f) and B.I.b.1. e) | | B.I.a.2.b) | | B.II.b.1 e/f) | |
| **WHO** | < 1 month | do (implement the change) and tell (inform the regulator) | 1-3 months | tell, wait for formal approval and then do | 3-6 months | tell, wait for formal approval and then do | 1-3 months | tell, wait for formal approval and then do |
| **Reference** (TRS 996, Annex 10, 2016; timelines based on Vx/Bx WHO reference) | Notification – no potential or minimal potential to have a negative impact on QSE | | Minor – potential to have a moderate or negative impact on QSE | | Major – significant potential to have a negative impact on QSE | | Minor – potential to have a moderate or negative impact on QSE | |

**BIOLOGICAL/VACCINE**

|  | **Specification change (tighten) to the drug substance or drug product** | | **Specification change (widen) to the drug substance or drug product** | | **Manufacturing change to the drug substance e.g. change to synthesis or additional manufacturing step** | | **New drug product formulation facility (new site)** | |
| --- | --- | --- | --- | --- | --- | --- | --- | --- |
|  | Time range of variation | Classification of change | Time range of variation | Classification of change | Time range of variation | Classification of change | Time range of variation | Classification of change |
| **EU** | < 1 month | do (implement the change) and tell (inform the regulator) | 3-6 months | tell, wait for formal approval and then do | 3-6 months | tell, wait for formal approval and then do | 3-6 months | tell, wait for formal approval and then do |
| **Reference** (2013/C 223/01) | B.I.b.1. b) and B.II.d.1 a) | | B.I.b.1. f) and B.I.b.1. e) | | B.I.a.2.c) | | B.II.b.1 c) | |
| **WHO** | < 1 month | do (implement the change) and tell (inform the regulator) | 1-3 months | tell, wait for formal approval and then do | 3-6 months | tell, wait for formal approval and then do | 1-3 months | tell, wait for formal approval and then do |
| **Reference** (TRS 1011, Annex 3, 2018 and TRS 993, Annex 4, 2015) | Bx – 22.g. and 55.g.  Vx – 18.h. and 46.i. | | Bx – 22.f. and 55.f.  Vx – 18.g. and 46.h. | | Bx – 7.a.  Vx – 4.a. | | Bx – 38.a.  Vx – 33.a. (conditions same as Bx) | |


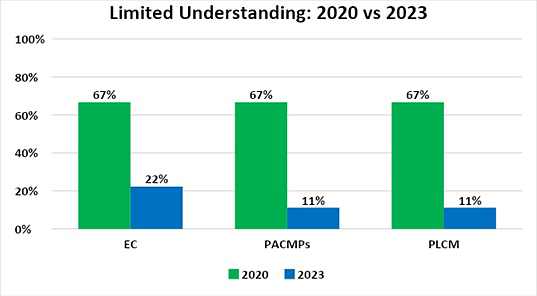
***Annex Figure:*** *Shows the difference between 2020 and 2023 on whether ‘Limited Understanding’ is hindering the implementation of ECs, PACMPs and PLCM.*

*Limitations and Challenges*

There were several limitations with the survey that are worth noting:

1. This is an industry perspective and we accept that regulators and other stakeholders may have a different perception. The approach is also dependent on the views of the participating companies and members in each of the EFPIA and IFPMA networks.
2. The survey did not examine the way the risk-based categorization is adhered to and applied; it only assessed if the respondent considered some type of risk-based categorization to be in place.
3. The analysis did not capture the impact of additional local requirements needed before submission and factors like company strategy and supply constraints which can further influence PAC timing.
4. We did not use statistical methodology to compare the patterns, only a visual comparison, therefore the true differences may not be apparent.
5. The use of reliance may be subjective. For example, use of a Certificate of Pharmaceutical Product (CPP) from a reference authority might, mistakenly, be considered reliance even if a further review is undertaken by the receiving agency. Also, we did not distinguish between permanent or temporary use of reliance e.g. to address a backlog in variations. Thus, it may be beneficial to understand better these factors in future surveys and get a more robust picture around the benefits and challenges related to the use of reliance.

A key challenge was the evolving legislation and landscape for PACs. We fixed the window for survey responses from April - June 2023 and asked respondents for a timeline and classification at this timepoint. Three common remarks from respondents were recorded during the survey.

1. In some cases it was difficult to assign parameters due to lack of guidance resulting in varying practice across member companies (we recorded one consensus response across the regulatory network).
2. A maximum of >18 months for the time to approval of a change was selected. Several respondents noted that timelines were even longer (>24 months) for some countries; these were recorded at the >18 month timepoint. For further surveys it may be helpful to include longer timelines to capture these outliers and show the extent of timelines faced by applicants.
3. Respondents faced changing requirements and regulations, thus we selected a narrow window to collect the results from respondents. Interestingly, we know of at least one case where the legislation significantly changed a month after the survey. This would have led to a more stringent classification and longer timeline than the benchmark but we maintained the original assessment to adhere to the time window of the survey.
